# Supplementary material for: Genetic editing of the virulence gene of Escherichia coli using the CRISPR system
Source: PeerJ. 2020 Apr 6;8:e8881. doi: 10.7717/peerj.8881 (PMC7144585; doi:10.7717/peerj.8881)
Supplement: Table S1 — Underline is the enzyme cleavage site, the italic is N20, and the double underline is the part of the pTarget plasmid. *Primers are from http://www.addgene.org/. [file peerj-08-8881-s001.docx]

**Table S1:**

**Primer sequences**

| Primer | Primer name | sequence | Primer position | function |
| --- | --- | --- | --- | --- |
| P1 | psgRNA-LT-Ⅰ-F | CTAGACTAGT*AAGCTTGGAGAGAAGAACCC*GTTTTAGAGCTAGAAATAGC | pTargetF 247-266 | Amplification of N20 (*eltⅠ*) + sgRNA |
| P2 | psgRNA- LT-Ⅰ-R | GGATAAAATGTGTAGTGAAAGTCGACTCTAGAGAATTCAAAAAAAGCACCG | *eltⅠ* 1199-1218  pTargetF 317-347 |  |
| P3 | LT-Ⅰ DonorL-F | TAGAGTCGACTTTCACTACACATTTTATCCTCG | pTargetF 338-347  *eltⅠ* 1199-1221 | Amplification of *eltⅠ* DonorL |
| P4 | LT-Ⅰ DonorL-R | AGTTGTTATATAGGCTCCTAGC CGAGGAAAACTTATATATCATACAA | *eltⅠ* 2507-2528  1311-1335 |  |
| P5 | LT-Ⅰ DonorR-F | GCTAGGAGCCTATATAACAACT | *eltⅠ* 2507-2528 | Amplification of *eltⅠ* DonorR |
| P6 | LT-Ⅰ DonorR-R | CCGCTCGAGTAACAGCTCCCTGTAGTGGA | *eltⅠ* 2619-2638 |  |
| P7 | LT-Ⅰ-F | CTAATAAAAATAATCAGGTTGCC | *eltⅠ* 1109-1131 | Identification primer |
| P8 | LT-Ⅰ-R | CCTCAGACCTGTCACATTTAT | *eltⅠ* 2746-2766 |  |
| P9 | psgRNA-STb-F | CTAGACTAGT*CAAATAATGGTTGCAGCAAA*GTTTTAGAGCTAGAAATAGC | pTargrtF 247-266 | Amplification of N20 (*eltⅡc1*) + sgRNA |
| P10 | psgRNA-STb-R | GTATTTACATGTCGACTCTAGAGAATTCAAAAAAAGCACCG | *estB* 287-296  pTargrtF 317-347 |  |
| P11 | STb DonorL-F | TAGAGTCGACATGTAAATACCTACAACGGGTGAT | pTargetF 338-347  *estB* 287-310 | Amplification of *estB* DonorL |
| P12 | STb DonorL-R | ATTGATAAAT AAATCACCTCAACCTTATTGCTAT | *estB* 674-683  *estB* 430-453 |  |
| P13 | STb DonorR-F | GAGGTGATTT ATTTATCAATAGCATTCAGCACC | *estB* 444-453  *estB* 674-696 | Amplification of *estB* DonorR |
| P14 | STb DonorR-R | CCGCTCGAGGCACCACTTAATTCCAAAGAAC | *estB* 878-899 |  |
| P15 | STb-F | CCCTCTCTTTTGCACTTCTTTC | *estB* 209-230 | Identification primer |
| P16 | STb-R | GGCACACTATTAACCAAACCAA | *estB* 947-968 |  |
| P17 | psgRNA-LT-Ⅱ-F_400_ | CTAGACTAGT*TCTTTTGGTGCGATAGAAGG*GTTTTAGAGCTAGAAATAGC | pTargetF 247-266 | Amplification of N20 (*eltⅡc1*) + sgRNA |
| P18 | psgRNA-LT-Ⅱ-R_400_ | GACAGGTTTGGTCGACTCTAGAGAATTCAAAAAAAGCACCG | *eltⅡc1* 218-227  pTargetF 317-347 |  |
| P19 | LT-Ⅱ DonorL-F_400_ | TAGAGTCGACCAAACCTGTCTCCGGATATT | pTargetF 338-347  *eltⅡc1* 218-237 | Amplification of *eltⅡc1* DonorL |
| P20 | LT-Ⅱ DonorL-R_400_ | CCAATGACTA TTTAATTTTTCACAACACCCT | *eltⅡc1* 1757-1766  601-621 |  |
| P21 | LT-Ⅱ DonorR-F_400_ | AAAAATTAAA TAGTCATTGGTGTTTTAGTTTTT | *eltⅡc1* 612-621  *eltⅡc1* 1757-1772 | Amplification of *eltⅡc1* DonorR |
| P22 | LT-Ⅱ DonorR-R_400_ | CCGCTCGAGCCAAATTGCTTAATGCTTTCT | *eltⅡc1* 2202-2222 |  |
| P23 | LT-Ⅱ-F1 | GTTCTGGGAAAAGAGGGAGG | *eltⅡc1* 31-50 | Identification primer |
| P24 | LT-Ⅱ-R1 | GGGGCGTGAACGTAATAGT | *eltⅡc1* 2735-2753 |  |
| P25 | psgRNA*-*STa-F | CTAGACTAGT*TGTTGTAATCCTGCCTGTGC*GTTTTAGAGCTAGAAATAGC | pTargetF 247-266 | Amplification of N20 (*estA*) + sgRNA |
| P26 | psgRNA-STa-R | GTACACTCGCGTCGACTCTAGAGAATTCAAAAAAAGCACCG | *estA* 555-564  pTargetF 317-347 |  |
| P27 | STa DonorL-F | TAGAGTCGACGCGAGTGTACCTCGACATATA | pTargetF 338-347  *estA* 174-194 | Amplification of *estA* DonorL |
| P28 | STa DonorL-R | TCTATGCTTT GTTACCTCCCGTCATGTTGT | *estA* 505-514  266-285 |  |
| P29 | STa DonorR F | GGGAGGTAAC AAAGCATAGAGGGAATCTTTAT | *estA* 276-285  505-526 | Amplification of *estA* DonorR |
| P30 | STa DonorR-R | CCGCTCGAGTGGAGTCATTACCTATAAAAGC | *estA* 584-605 |  |
| P31 | STa-F1 | CATTACCCGAACAAGAAAAG | *estA* 60-79 | Identification primer |
| P32 | STa -R2 | TGAACATAAAACACTATCAATAAGT | *estA* 606-630 |  |
| P33 | psgRNA-K88-F | GGACTAGT*GCCGGTGTGTTCGGGAAAGG*GTTTTAGAGCTAGAAATAGC | pTargetF 247-266 | Amplification of N20 (*faeG*) + sgRNA |
| P34 | psgRNA-K88-R | TCCACAAATAATAAACCTAGAGAATTCAAAAAAAGCA | *faeG* 60-75  pTargetF 320-340 |  |
| P35 | K88 DonorL-F | TGCTTTTTTTGAATTCTCTAGGTTTATTATTTGTGGA | pTargetF 320-340  *faeG* 60-75 | Amplification of *faeG* DonorL |
| P36 | K88 DonorL-R | TTACTCTTTGAATCTGTCC TCCATTAAGACCTGTACC | *faeG* 736-755  *faeG* 245-262 |  |
| P37 | K88 DonorR-F | GGTACAGGTCTTAATGGA GGACAGATTCAAAGAGTAA | *faeG* 245-262  737-755 | Amplification of *faeG* DonorR |
| P38 | K88 DonorR-R | CCCTCGAGGTTGCCTCAATAGTCTGACCGTT | *faeG* 860-882 |  |
| P39 | K88-F | GGGATGGTTTTACGGTAATTC | *faeG* 13-33 | Identification primer |
| P40 | K88-R | ATTGCTACGTTCAGCGGAGCG | *faeG* 919-939 |  |
| P41 | STb DonorL-R-STa | TCGGGTAATG AAATCACCTCAACCTTATTGCTAT | *estA* 60-69  *estB* 430-453 | Amplification of *estB* DonorL with STb DonorL-F |
| P42 | r-STa F1 | GAGGTGATTT CATTACCCGAACAAGAAAAG | *estB* 444-453  *estA* 60-79 | Amplification of the fragment of the inserted *estA* |
| P43 | r-STa R1 | ATTGATAAAT TTCTCAGCACCAATACATATAAT | *estA* 542-564  *estB* 674-683 |  |
| P44 | STb DonorR-F-STa | GTGCTGAGAA ATTTATCAATAGCATTCAGCACC | *estA* 555-564  *estB* 674-696 | Amplification of *estB* DonorR with STb DonorR-R |
| P45 | psgRNA-LT-Ⅱ-R1 | GAGTCTGCTCTAAAGAAATCGCTAGAGAATTCAAAAAAAGCA | *eltⅡc1* 678-698  pTargetF 320-340 | Amplification of N20 (*eltⅡc1*) + sgRNA with psgRNA-LT-Ⅱ-F_400_ |
| P46 | LT-Ⅱ DonorL-F1 | TGCTTTTTTTGAATTCTCTAG CGATTTCTTTAGAGCAGACTC | pTargetF 320-340  *eltⅡc1* 678-698 | Amplification of *eltⅡc1* DonorL |
| P47 | LT-Ⅱ DonorL-R1-K88 | GCTCCTTCATAGTCAGTAAAT GGACTATACCGCCCTAACAC | *faeG* 409-429  *eltⅡc1* 967-986 |  |
| P48 | rK88-F | GTGTTAGGGCGGTATAGTCC ATTTACTGACTATGAAGGAGC | *eltⅡc1* 967-986  *faeG* 409-429 | Amplification of the fragment of the inserted *faeG* |
| P49 | rK88-R | ATCCTTAAAAGTTTTACTTACG AAACCACCATAAAAGATAGCG | *eltⅡc1* 1462-1483  *faeG* 622-642 |  |
| P50 | LT-Ⅱ DonorR-F1-K88 | CGCTATCTTTTATGGTGGTTT CGTAAGTAAAACTTTTAAGGAT | *faeG* 622-642  *eltⅡc1*  1462-1485 | Amplification of *eltⅡc1* DonorR |
| P51 | LT-Ⅱ DonorR-R1 | CCTCGAGCGGTAGGTTTCCACATAGT | *eltⅡc1*  1870-1888 |  |
| P52 | LT-Ⅱ DonorL-R2 | GAGCAAATGCACTCCATGGGAGCTCTCGCCATGC | *eltⅡc1*  1186-1219 | Amplification of *eltⅡc1* DonorL with LT-Ⅱ DonorL-F1 |
| P53 | LT-Ⅱ DonorR-F2 | GCATGGCGAGAGCTCCCATGGAGTGCATTTGCTC | *eltⅡc1*  1186-1219 | Amplification of *eltⅡc1* DonorR with LT-Ⅱ DonorR-R1 |
| P54 | LT-Ⅱ-F2 | AATGGATAGGGTGTTGTG | *eltⅡc1* 594-611 | Identification primer |
| P55 | LT-Ⅱ-R2 | TGCTCAGGGCTGTGGGTCCT | *eltⅡc1*  2167-2186 |  |
| P56 | psgRNA-LT-Ⅱ-R_300_ | CTTTTGATTAGTCGACTCTAGAGAATTCAAAAAAAGCACCG | *eltⅡc1* 318-327  pTargetF 317-347 | Amplification of N20 (*eltⅡc1*) + sgRNA with psgRNA-LT-Ⅱ-F_400_ |
| P57 | LT-Ⅱ DonorL-F_300_ | TAGAGTCGACTAATCAAAAGGGGACGTTAT | pTargetF 338-347  *eltⅡc1* 318-337 | Amplification of *eltⅡc1* Donor |
| P58 | LT-Ⅱ DonorR-R_300_ | CCGCTCGAGATGAATAAAAAAGCTCACCAT | *eltⅡc1*  2067-2087 |  |
| P59 | psgRNA-LT-Ⅱ-R_200_ | GGCGCGTAACGTCGACTCTAGAGAATTCAAAAAAAGCACCG | *eltⅡc1* 387-396  pTargetF 317-347 | Amplification of N20 (*eltⅡc1*) + sgRNA with psgRNA-LT-Ⅱ-F_400_ |
| P60 | LT-Ⅱ DonorL-F_200_ | TAGAGTCGACGTTACGCGCCTATCTGTCG | pTargetF 338-347  *eltⅡc1* 387-405 | Amplification of *eltⅡc1* Donor |
| P61 | LT-Ⅱ DonorR-R_200_ | CCGCTCGAGTCCTTACCGTTAGTTTCAATAC | *eltⅡc1*  1937-1958 |  |
| P62 | psgRNA-LT-Ⅱ-R_100_ | TGACTGGTTGGTCGACTCTAGAGAATTCAAAAAAAGCACCG | *eltⅡc1* 498-507  pTargetF 317-347 | Amplification of N20 (*eltⅡc1*) + sgRNA with psgRNA-LT-Ⅱ-F_400_ |
| P63 | LT-Ⅱ DonorL-F_100_ | TAGAGTCGACCAACCAGTCATTTGTCTACG | pTargetF 338-347  *eltⅡc1* 498-517 | Amplification of *eltⅡc1* Donor |
| P64 | LT-Ⅱ DonorR-R_100_ | CCGCTCGAGTTTAAATTAGGAACGTGTCAA | *eltⅡc1*  1839-1859 |  |
| P65 | psgRNA-LT-Ⅱ-R_50_ | ATTAAAGGTGGTCGACTCTAGAGAATTCAAAAAAAGCACCG | *eltⅡc1* 573-582  pTargetF 317-347 | Amplification of N20 (*eltⅡc1*) + sgRNA with psgRNA-LT-Ⅱ-F_400_ |
| P66 | LT-Ⅱ DonorL-F_50_ | TAGAGTCGACCACCTTTAATAATATTTTTTAAATG | pTargetF 338-347  *eltⅡc1* 573-597 | Amplification of *eltⅡc1* Donor |
| P67 | LT-Ⅱ DonorR-R_50_ | CCGCTCGAGAAATACAGAATAAAATTGAATGAA | *eltⅡc1*  1782-1805 |  |
| P68 | L4440* | AGCGAGTCAGTGAGCGAG | pTargetF 98-115 | Identification primer |
| P69 | pTarget-R | CCTCTGATAGTTGAGTCGATACTTC | pTargetF 529-553 |  |
| P70 | LacI-R* | GGCATACTCTGCGACATCGT | pCas 1448-1467 | Identification primer |
| P71 | pCasR1 | CCTTTTGTAATACTGCGGAACTG | pCas 2930-2952 |  |

^a^ The underline is the enzyme cleavage site, the italic is N20, and the double underline is the part of the pTarget plasmid.

*Primers are from <http://www.addgene.org/>.
