# Supplementary figures and images for: Genetic editing of the virulence gene of Escherichia coli using the CRISPR system
Source: PeerJ. 2020 Apr 6;8:e8881. doi: 10.7717/peerj.8881 (PMC7144585; doi:10.7717/peerj.8881)

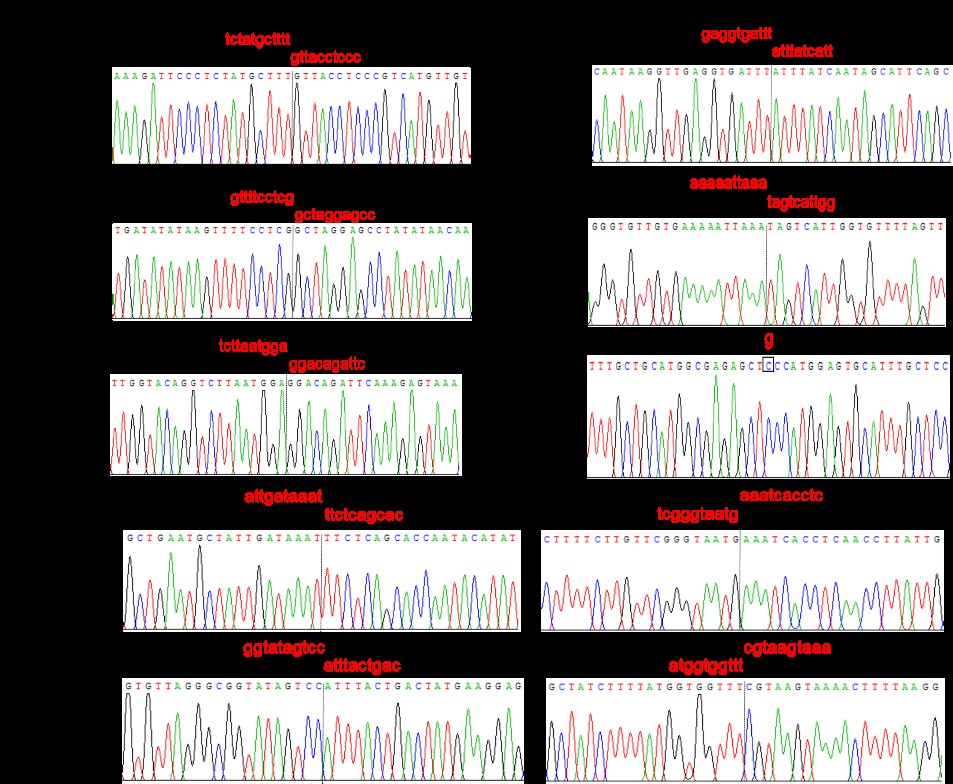

Supplement: Figure S1 [file peerj-08-8881-s002.jpg]

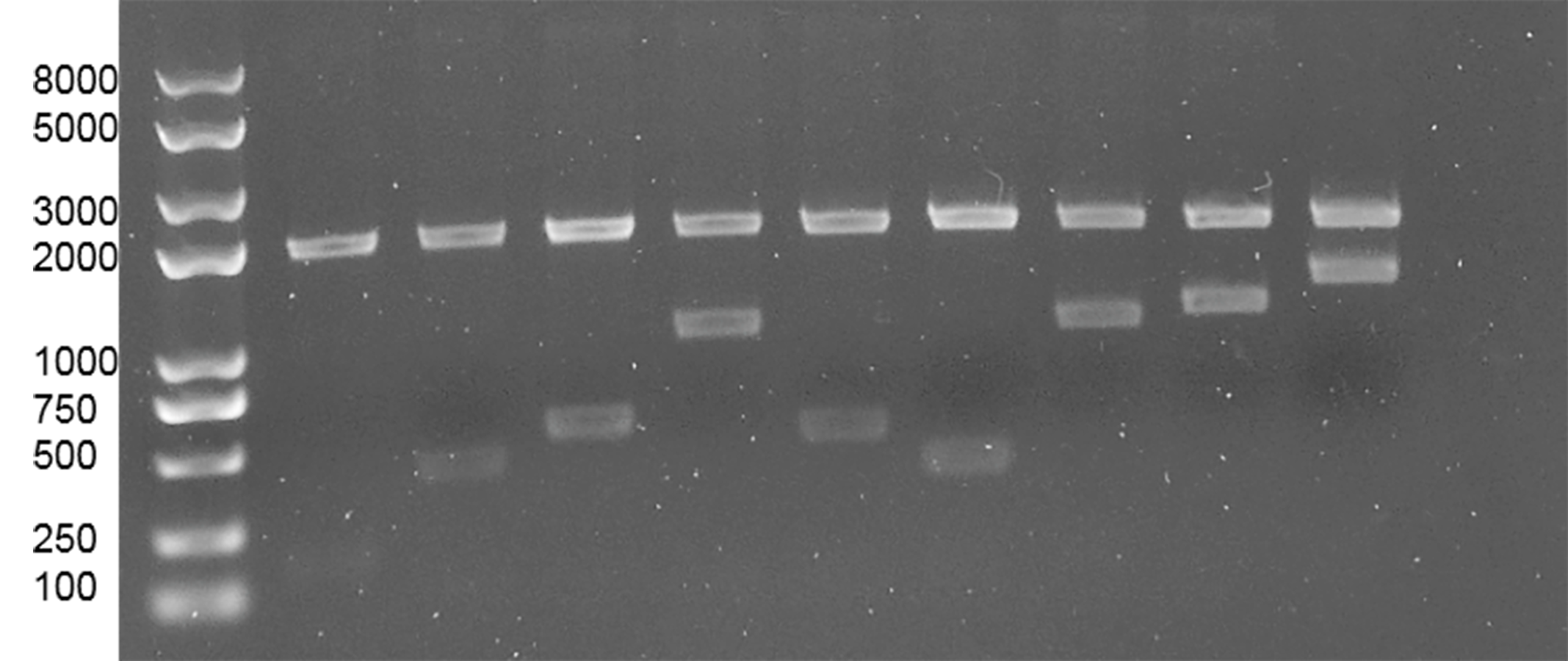

Supplement: Figure S2 — From left to right are: pTarget, pTarget-Delt, pTarget-Δstb, pTarget-stb::estA, pTarget-ΔfaeG, pTarget-ΔestA, pTarget-ΔeltIIc1, pTarget-ΔeltIIc1::faeG, pTarget-ΔfaeG:: eltIIc1193 [file peerj-08-8881-s003.png]
