# Supplementary material for: Genetic editing of the virulence gene of Escherichia coli using the CRISPR system
Source: PeerJ. 2020 Apr 6;8:e8881. doi: 10.7717/peerj.8881 (PMC7144585; doi:10.7717/peerj.8881)
Supplement: Figure S3 [file peerj-08-8881-s004.docx]

The gene-deletion ETECs were tested in vivo and in vitro whether their toxicity changed.

LT-I enterotoxin was tested by rabbit intestinal loop ligation test. LT-I enterotoxin extraction: *E. coli* O141: K85, *E. coli* O141: K85Δ*estB*, and *E. coli* O141: K85Δ*estB*Δ*eltI* were cultured at an OD_600_ of 1.0. Take 1 ml of the bacterial solution and add it to 100 mL of CAYE liquid medium. After shaking culture at 37 °C for 18h, the culture was centrifuged at 10,000 r/min at 4 °C for 15 min. The cell pellet was resuspended in PBS to a final concentration of 0.1 g/mL. Polymyxin B was added to a final concentration of 2.0 × 10^7^ U/L. The above mixed solution was immersed in a 35 °C water bath for 18 hours, and shaken several times to fully disperse the bacteria. The above mixed solution was centrifuged at 5,000 r/min for 30 minutes. The supernatant was the polymyxin B extract. After adding ammonium sulfate to the supernatant to a saturation of 80 %, it was left at 4 °C overnight. After centrifugation at 10,000 r/min for 15 minutes at 4 °C, the pellet was suspended in 10 mL PBS. After dialysis with PBS, it was filtered through a 0.22 μm filter. 2 kg rabbits were fasted for 48 hours before surgery. After anesthesia, the abdomen was cut to expose the ileum. A surgical ligature was used for segmental ligation from the blind end to the cardiac end of the ileocecal junction. Each segment was ligated 5 cm as the test segment. 1mL of prepared enterotoxin solution was injected into each intestine. 1 mL CAYE medium was injected as the negative control. After all injections, the incision was sutured. Experimental rabbits were euthanized after 18 h. Dissect and remove all ligated segments. Then measure the length of each intestinal segment and the amount of fluid in the intestinal cavity. The index measured in this test is the amount of fluid in the intestine per cm. The positive result was that the volume of fluid in 1 cm intestine was more than 1 mL. It showed that the tested strain could produce heat-sensitive enterotoxin. There was no effusion in the intestine segment injected with CAYE. The intestinal segments injected with LT-I enterotoxin were swollen and filled. *E. coli* O141: K85Δ*estB* extract was injected into the intestine to produce a large amount of fluid. *E. coli* O141: K85Δ*estB*Δ*eltI* extract was injected, and there was no fluid in the intestine, which was the same as the control group (Figure S3 B). This showed that *E. coli* O141: K85 can produce LT-I enterotoxin. LT-I enterotoxin would no longer be produced after gene knockout. STB enterotoxin had no obvious effect on intestinal effusion. Figure S3 A showed the ratio of the amount of intestinal effusion to the length of the intestinal segment. It could be seen that the intestinal fluid volume per cm of intestinal segment injected with *E. coli* O141: K85 is greater than 1 mL. There was no significant fluid accumulation in the intestine injected with *E. coli* O141:K85Δ*estB*Δ*eltI*.

The suckling mouse assay was used to test the ability of *E. coli* O142 and *E. coli* O142Δ*estA* to produce STa enterotoxin. STa enterotoxin extract: the strain was streaked on a Mac Conkey Agar Medium and cultured at 37 °C for 18 h. A single colony was picked into 50 mL Honda Toxin-Producing Broth medium and incubate at 37 °C for 48 h. The culture was centrifuged at 15,000 r/min for 15 min, and the supernatant was sterilized by filtration through a 0.22 μm filter. 4-day-old BALB/c suckling mice were administrated with 100 μL of the culture. They were sacrificed 4 hours later. Calculate the ratio of intestinal weight to rest corpse weight of suckling mice. The ratio greater than 0.090 was defined as STa toxin positive. The ratios of mice in *E. coli* O142 group were greater than 0.090. The ratios of mice in *E. coli* O142Δ*estA* group were about 0.05 (Figure S3 C). It means that STa enterotoxin is not produced after knocking out the *estA* gene.

Y-1 cytotoxicity test was used to test the ability of *E.coli* DN1502, *E.coli* DN1502Δ*eltII* to produce LT-II enterotoxin. A single colony was picked into 50 mL Honda Toxin-Producing Broth medium and incubate at 37 °C for 24 h. The produced LT-II toxin is secreted into the culture medium. After centrifugation, the supernatant was collected and filtered to sterilize. *E. coli* DN1502 secretes LT-II enterotoxin. 9 hours after LT-II enterotoxin infected Y-1 cells, Y-1 cells showed obvious lesions. The diseased cells turned round from irregular "Y" shape (Figure S3 D1). *E.coli* DN1502Δ*eltII* supernatant infected group was the same as normal cells, without cell cytopathy (Figure S3 D2-3).


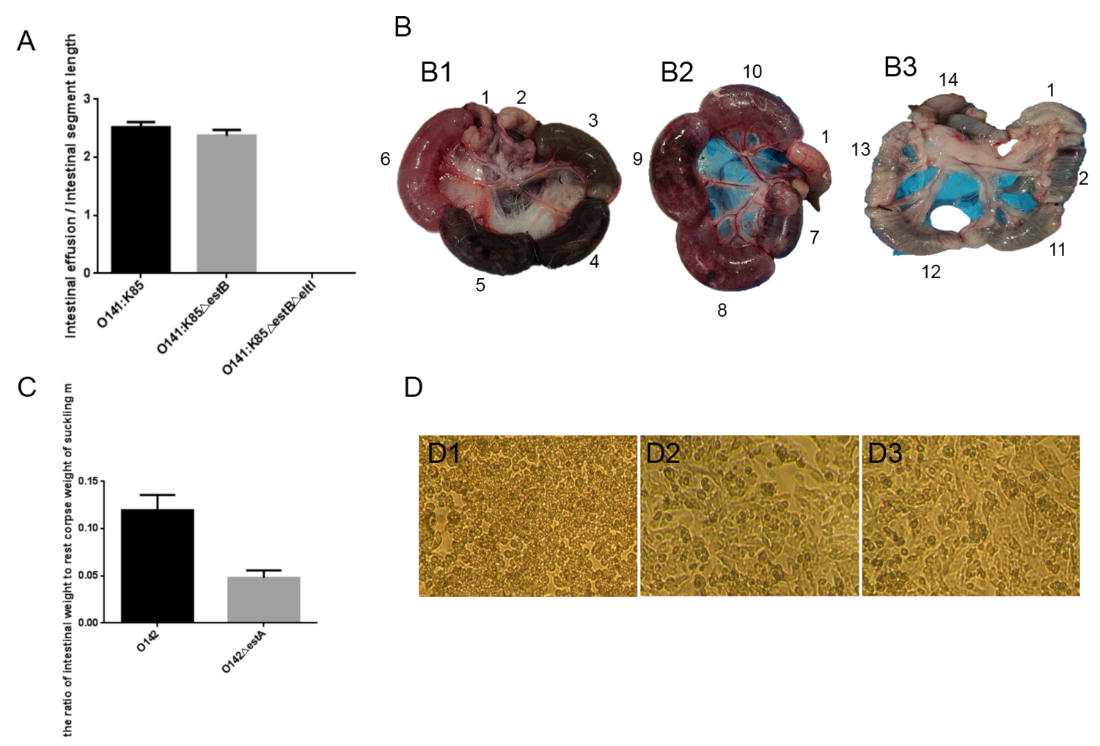


Figure S3. Toxic effects of ETEC enterotoxins

A: Intestinal effusion/Intestinal segment length.

B: The rabbit intestinal-loop assays for detection of LT-I. 1-2 was injected with CAYE medium. 3-6 was injected with LT enterotoxin extracted from *E. coli* O141: K85. 7-10 was injected with *E. coli* O141: K85Δ*estB* extract. 11-14 was injected with *E. coli* O141: K85Δ*estB*Δ*eltI* extract.

C: The ratio of intestinal weight to rest corpse weight of suckling mice.

D: Determination of LT-II enterotoxin on Y-1 cytotoxicity. D1: Infected Y-1 cells with LT-II enterotoxin extracted from *E. coli* DN1502. D2: Infected Y-1 cells with *E. coli* DN1502Δ*eltII* extract. D3: Normal Y-1 cells. (Monolayers were observed by microscopy and photographed at x 100)
