# Supplementary material for: Genetic editing of the virulence gene of Escherichia coli using the CRISPR system
Source: PeerJ. 2020 Apr 6;8:e8881. doi: 10.7717/peerj.8881 (PMC7144585; doi:10.7717/peerj.8881)
Supplement: Supplemental Information 1 [file peerj-08-8881-s005.docx]

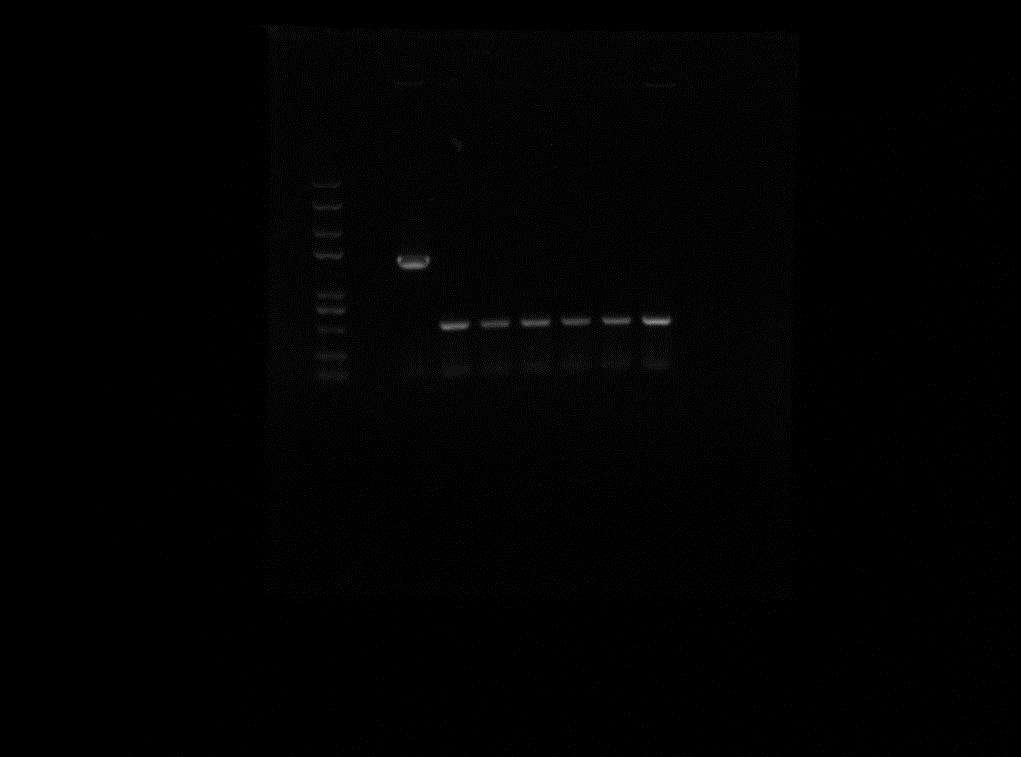


C83903 eltI knock off


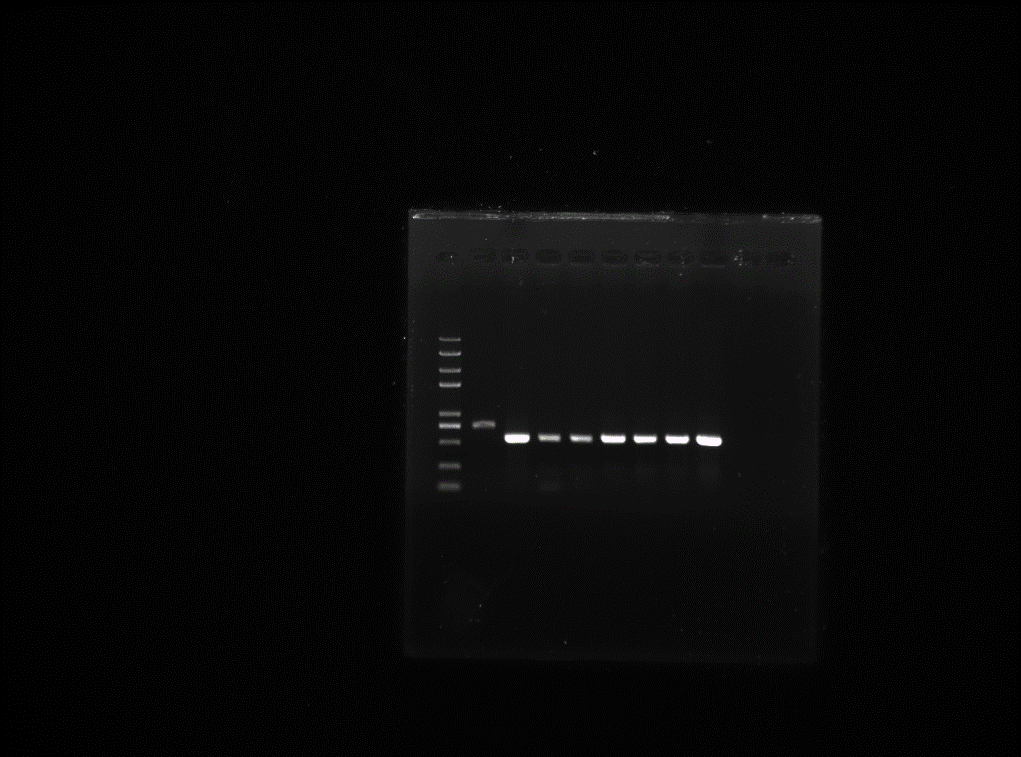


C83903 estb knock off


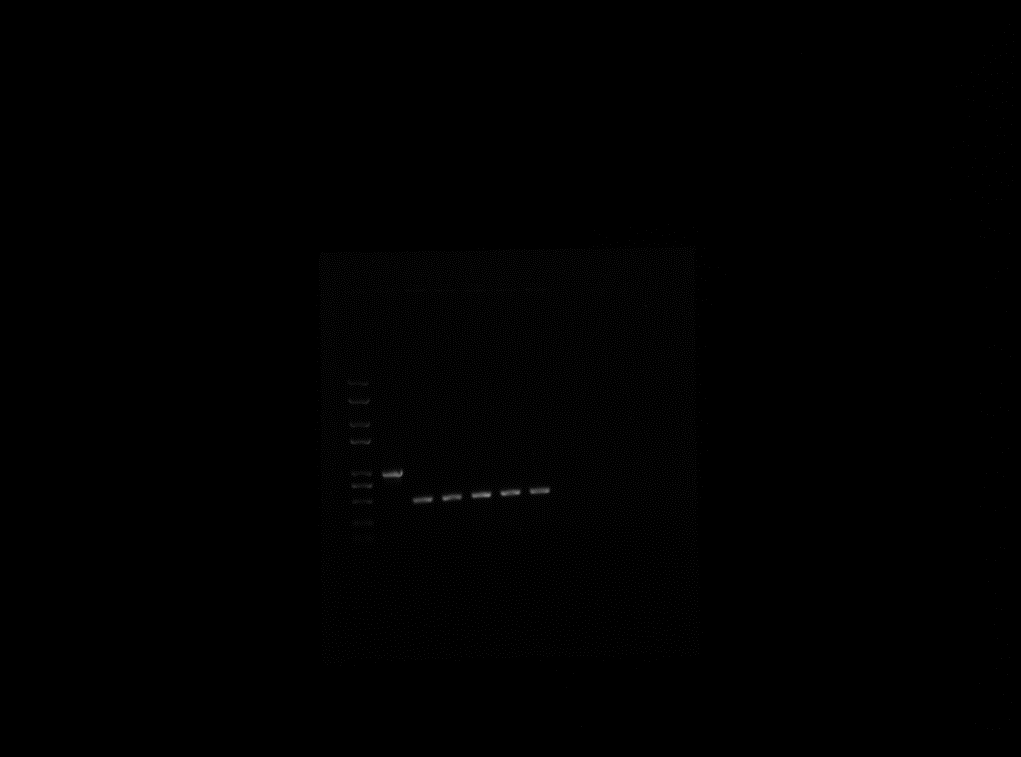


C83903 K88(faeG) knock off


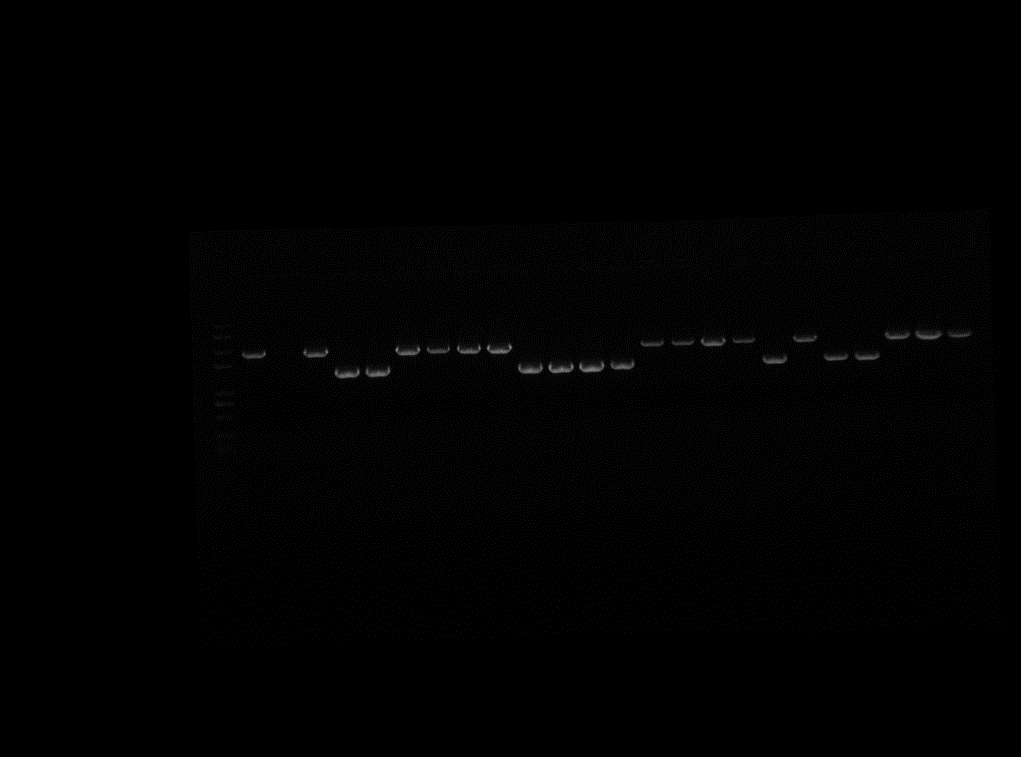


DN1502 eltII knock off


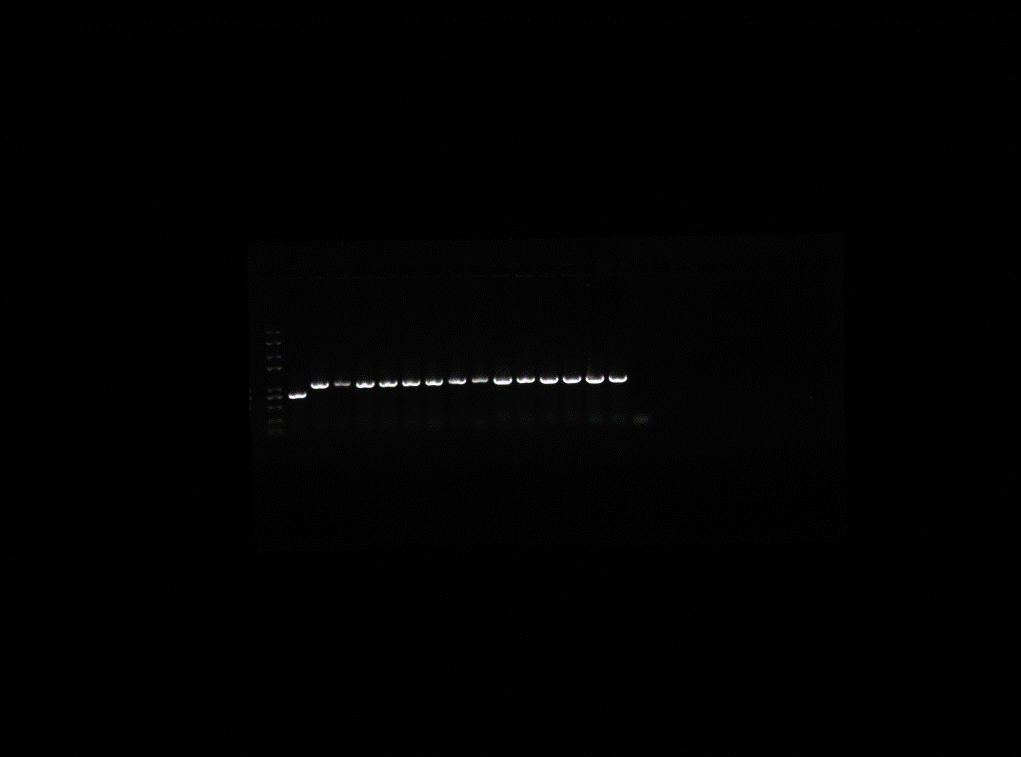


C83903 esta was inserted into estb


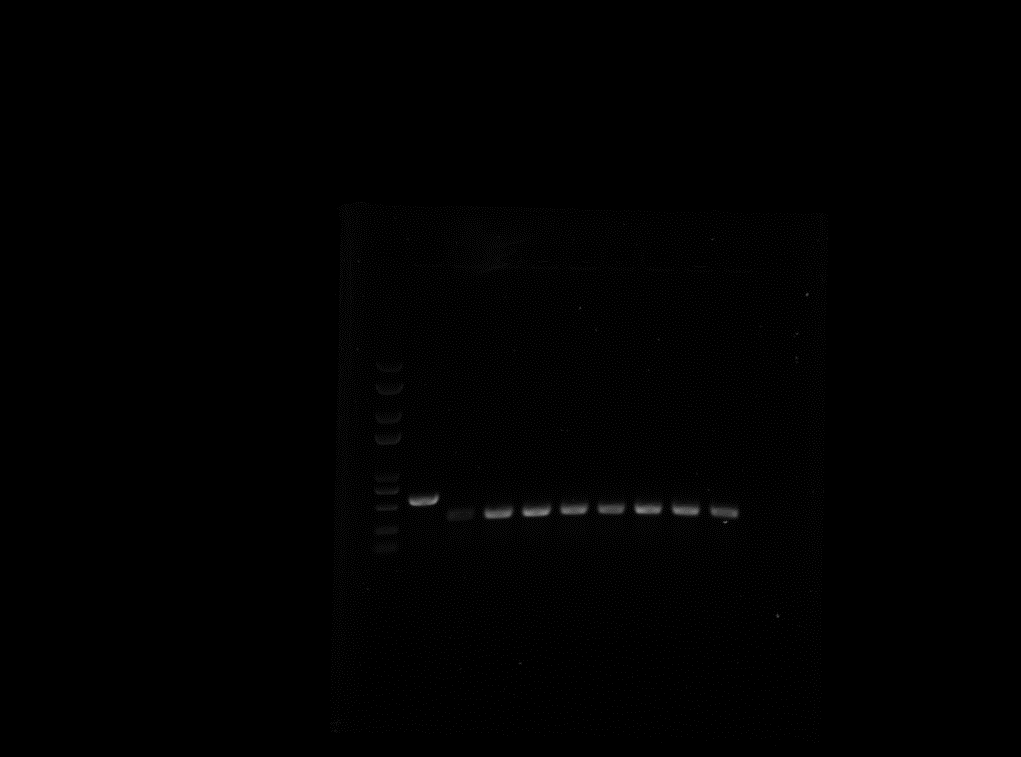


O142 esta knock off


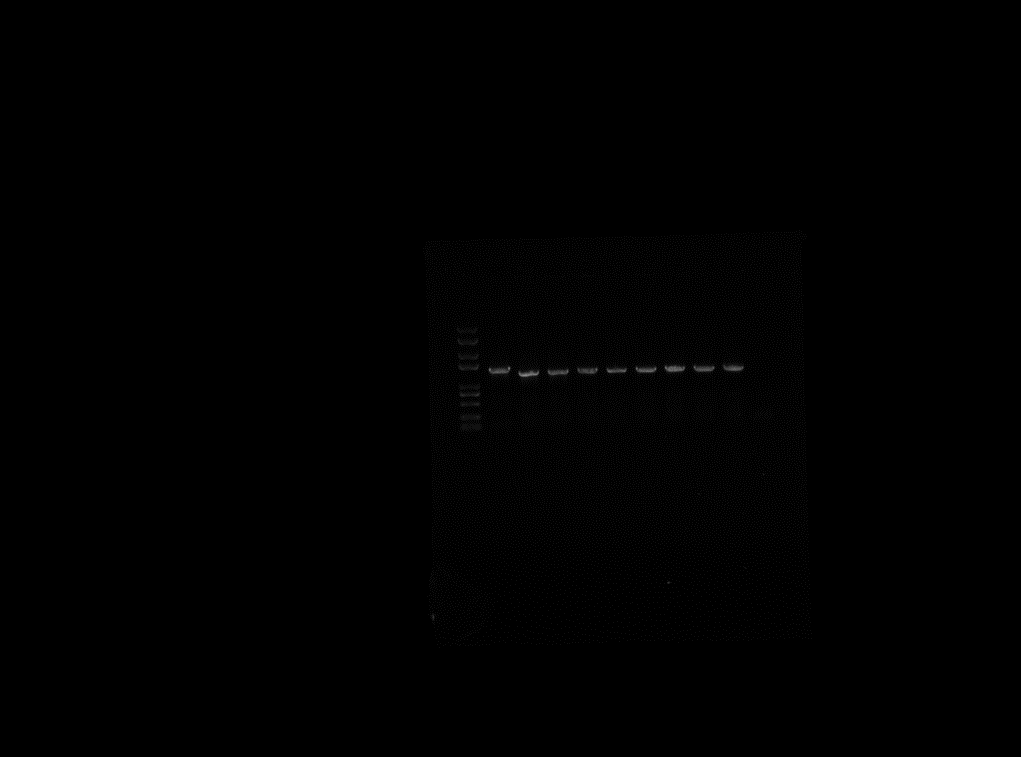


DN1502 the insertion(K88 faeG) of eltII


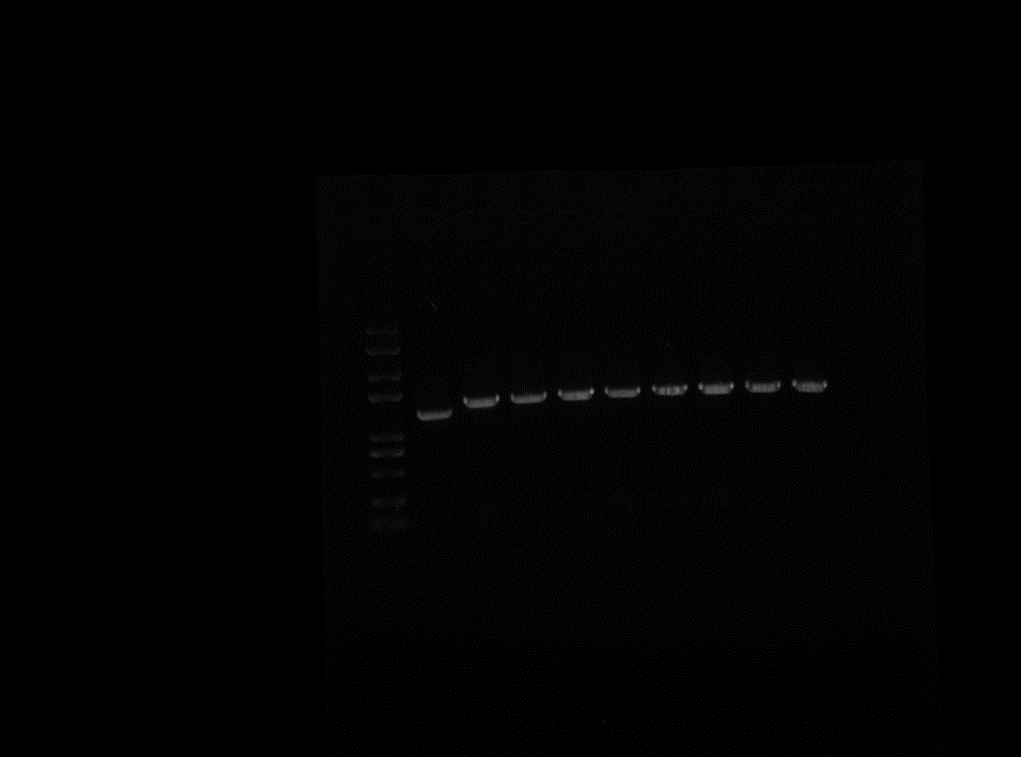


DN1502 the mutation of eltII


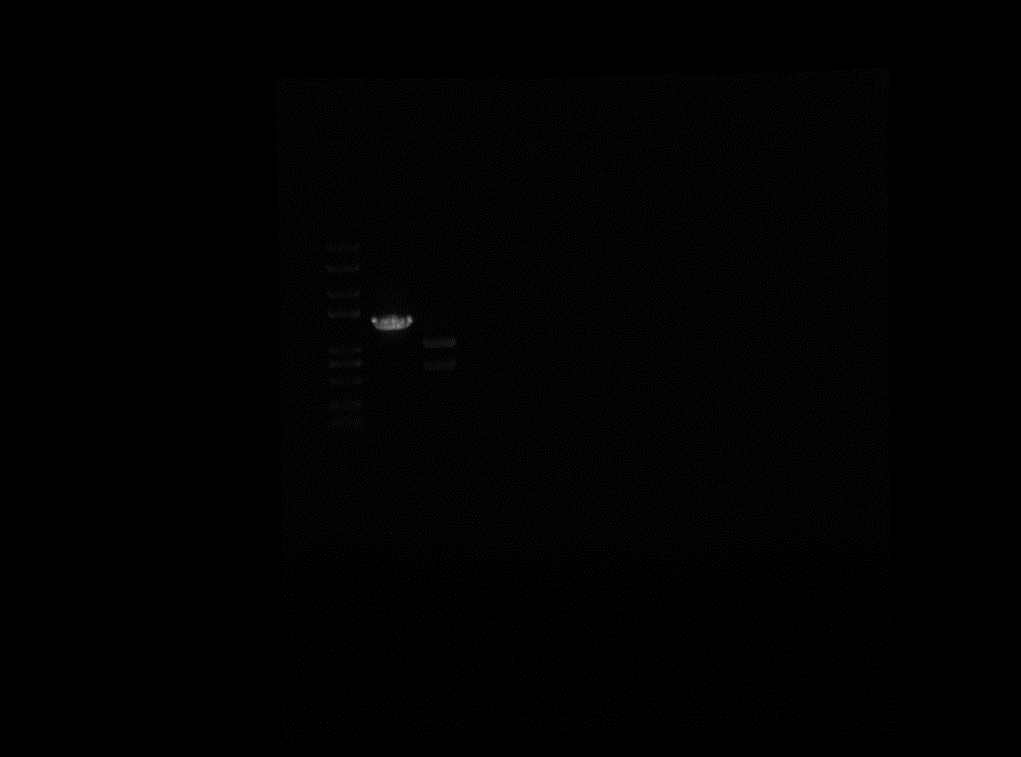


DN1502 the mutation and digestion of eltII


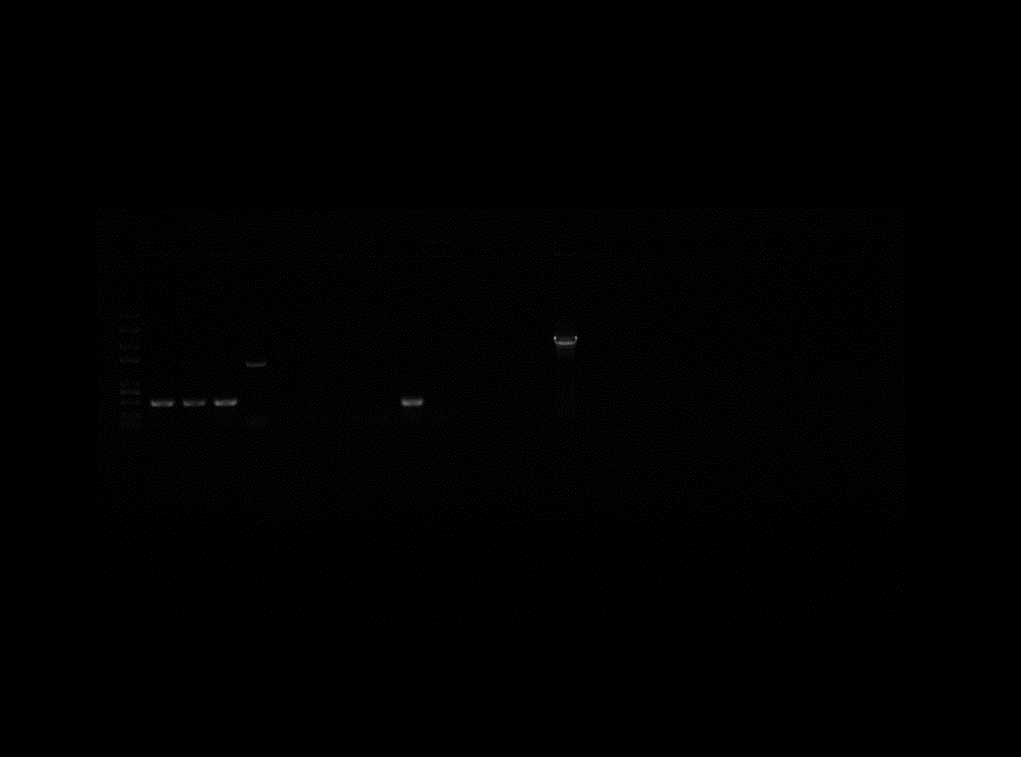


C83903 eltI knock off and cure plasmid


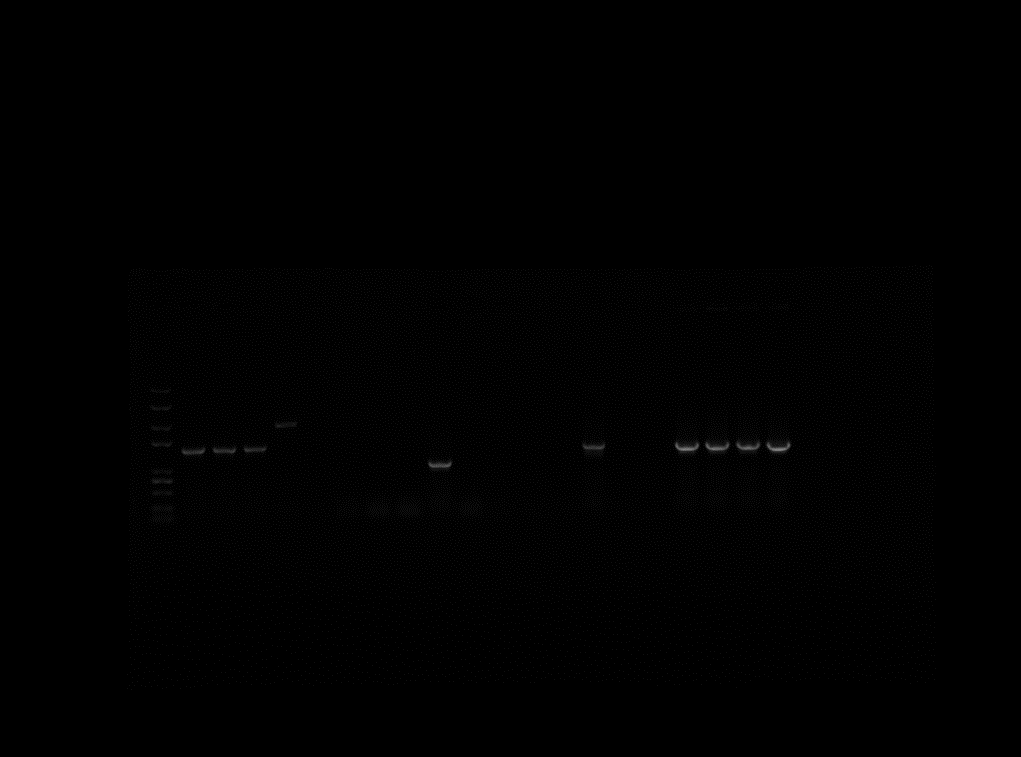


DN1502 after knocking off eltII, the plasmid cure


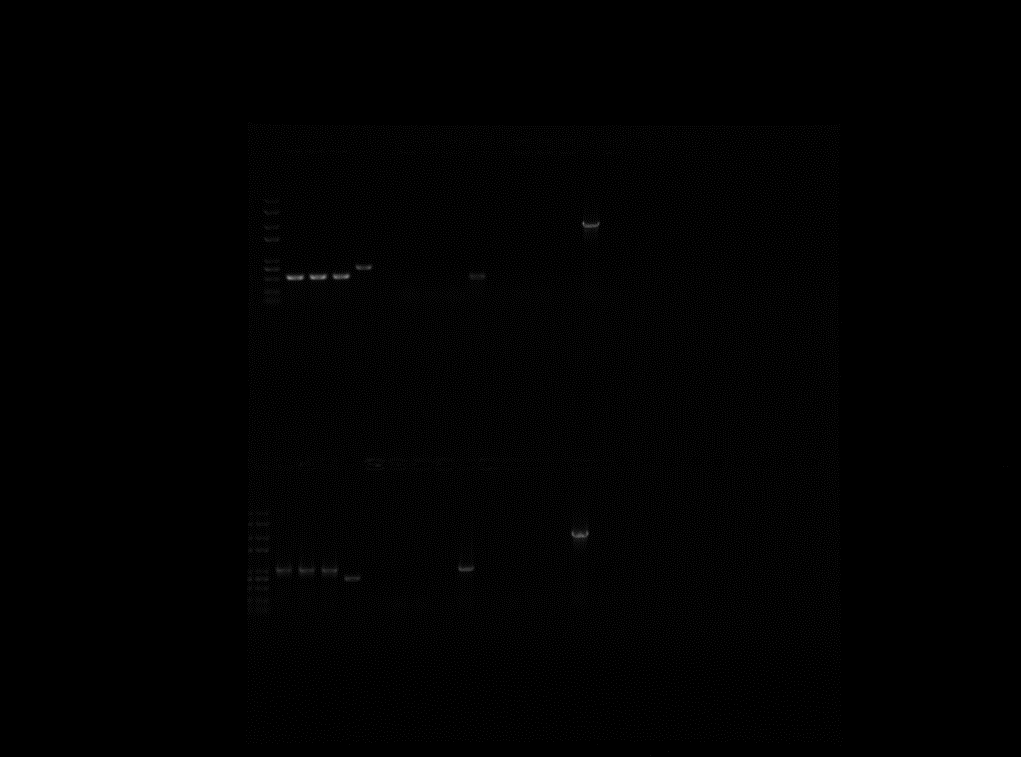


C83903 estb knock off and cure plasmid, estb inserted esta and plasmid cure


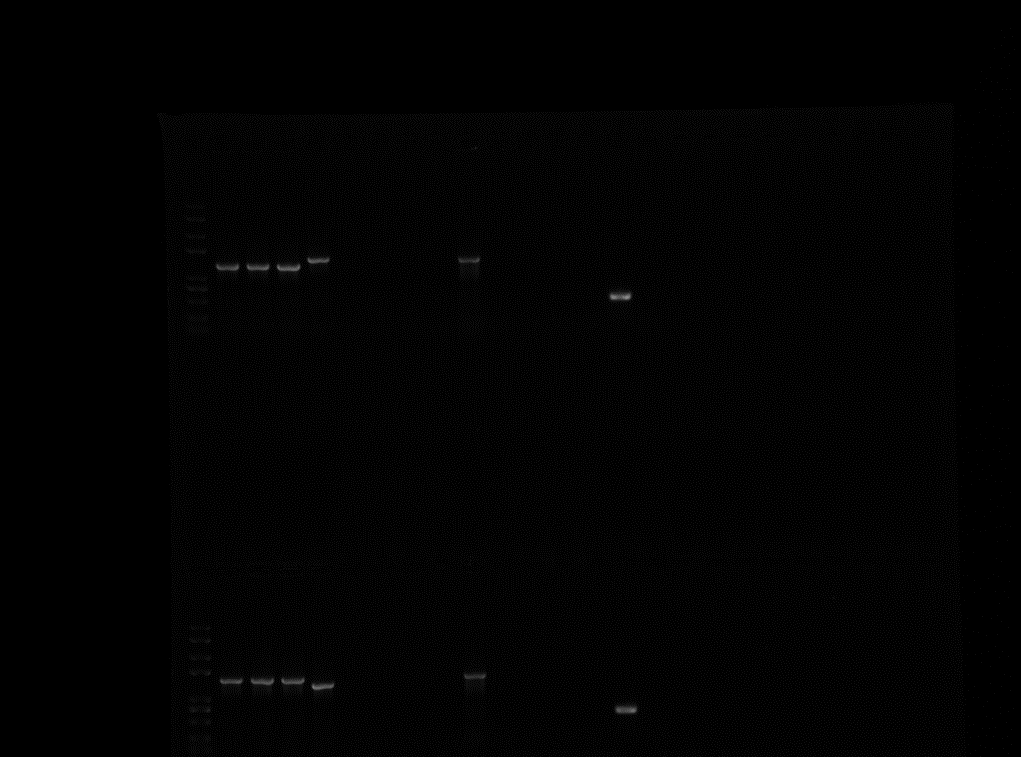


DN1502 the mutation and insertion(K88 faeG) of eltII and plasmid cure


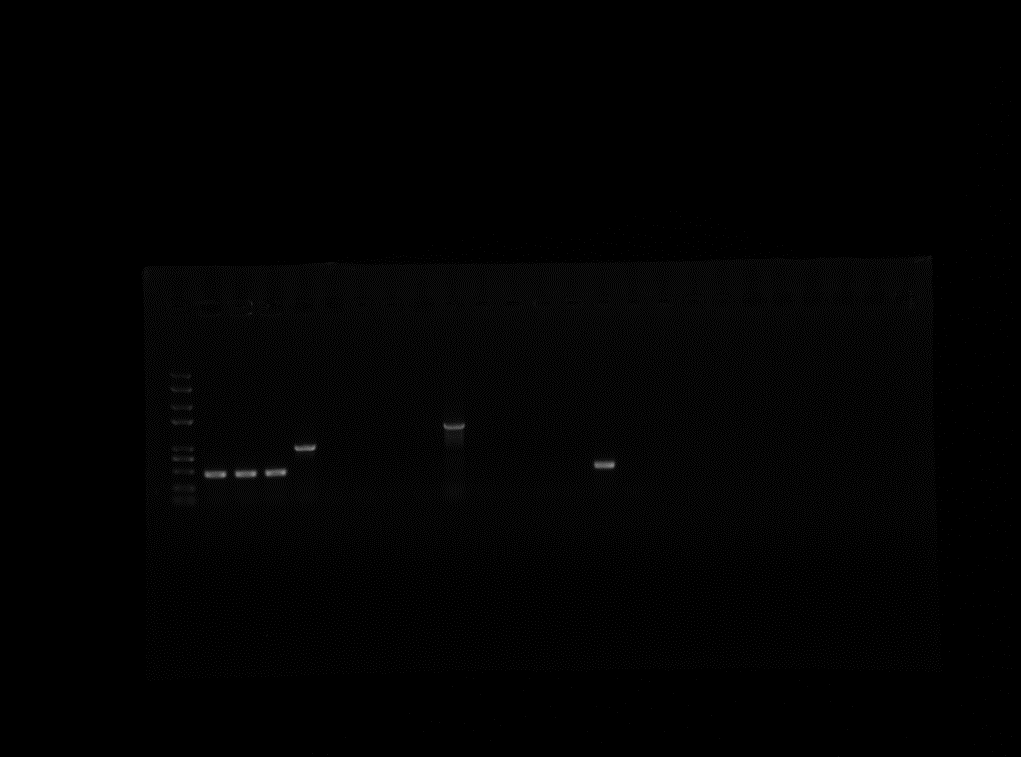


C83903 K88(faeG) knock off and plasmid cure


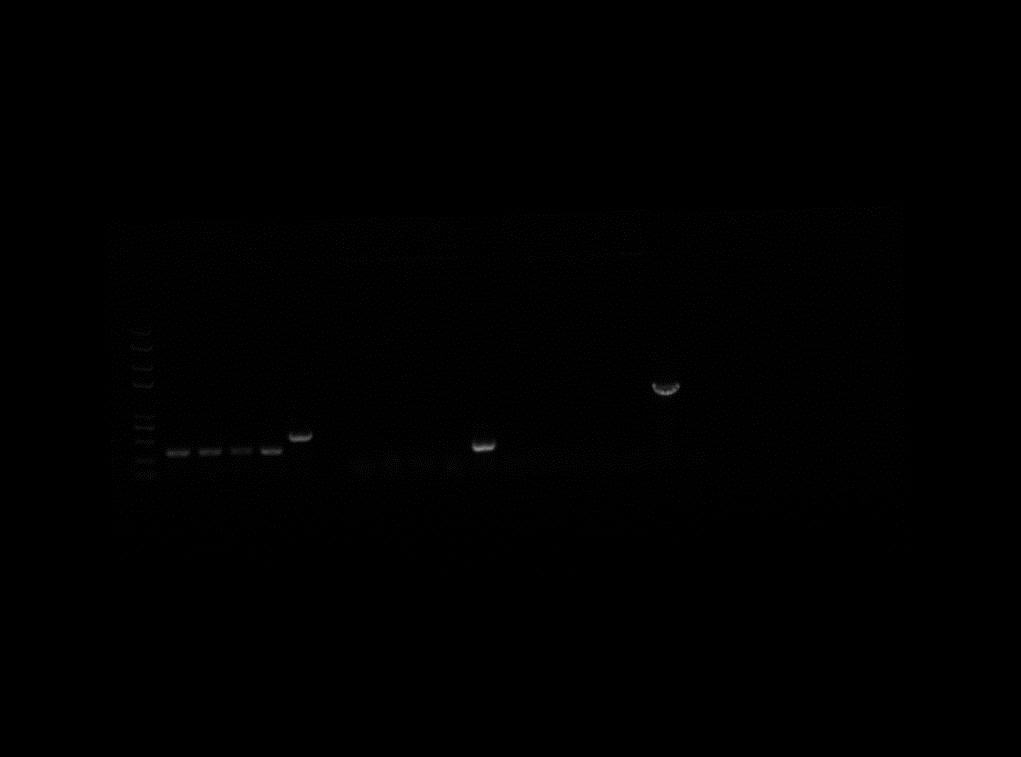


O142 esta knock off and cure plasmid

The raw figures were captured by alphamager HP.（Protein Simple, California, USA）

The figures appeared in manuscript was enhanced and clipped by photoshop CC 2018.
